# Engineered 3D-Printed Polyvinyl Alcohol Scaffolds Incorporating β-Tricalcium Phosphate and Icariin Induce Bone Regeneration in Rat Skull Defect Model

**DOI:** 10.3390/molecules27144535

**Published:** 2022-07-15

**Authors:** Zhimin Xu, Yidan Sun, Huanyan Dai, Yujie Ma, Han Bing

**Affiliations:** Department of Oral and Maxillofacial Surgery, School and Hospital of Stomatology, Jilin University, Changchun 130021, China; xuzhimin@jlu.edu.cn (Z.X.); sunyd18@mails.jlu.edu.cn (Y.S.); daihy20@mails.jlu.edu.cn (H.D.); mayj20@mails.jlu.edu.cn (Y.M.)

**Keywords:** three-dimensional printing, polyvinyl alcohol, icariin, β-tricalcium phosphate, skull base defects

## Abstract

The skull defects are challenging to self-heal, and autologous bone graft repair has numerous drawbacks. The scaffolds for the rapid and effective repair of skull defects have become an important research topic. In this study, polyvinyl alcohol (PVA)/β-tricalcium phosphate(β-TCP) composite scaffolds containing icariin (ICA) were prepared through direct-ink three-dimensional (3D) printing technology. β-TCP in the composite scaffold had osteoconductive capability, and the ICA molecule had osteoinductive capacity. The β-TCP and ICA components in the composite scaffold can enhance the capability to repair skull defects. We show that ICA exhibited a slow-release behaviour within 80 days. This behaviour helped the scaffold to continuously stimulate the formation of new bone. The results of in vitro cell compatibility experiments showed that the addition of ICA molecules contributed to the adhesion and proliferation of MC-3T3-E1 cells. The level of alkaline phosphatase secretion demonstrated that the slow release of ICA can promote the osteogenic differentiation of MC-3T3-E1 cells. The introduction of ICA molecules accelerated the in situ bone regeneration in in vivo. It is concluded that the 3D-printed PVA scaffold with β-TCP and ICA has a wide range of potential applications in the field of skull defect treatment.

## 1. Introduction

Infections, malignancies, injuries, and degenerative diseases such as osteoarthritis and rheumatoid arthritis cause serious bone defect issues and substantial incapacity in individuals all over the world [1]. It also has a significant negative impact on one’s usual quality of life and health [2]. Following that, successful therapies are required, as is bone regeneration management. Although bone tissue can heal itself after an injury, it is unlikely to do so when the damage is severe [1]. Bone grafts, including autografts and allografts, have been used to treat patients with standardised treatment for bone defects [3,4,5]. Including the immunological rejection, allografts have several drawbacks [6,7]. Innovative strategies for mending bone abnormalities are therefore required to avoid such constraints. Bone tissue engineering is a new method of treating bone abnormalities [8]. One of the new innovative strategies is the 3D printing. The advantages, current limitations, and future directions of 3D printing in animal orthopaedics has been discussed in a very recent article [9], and in a book chapter published recently [10]. The design and development of the craniofacial scaffolds, including material choices, scaffold fabrication workflows, and the mechanical, structural, and biological considerations impacting scaffold application and function have been discussed in a recent article [11]. The fabrication of a fully 3D-printed tunable lens with an inhomogeneous structure has been described by a group of researchers [12]. Similarly, with the advancement of medical technology in recent years, computed tomography (CT) has been able to scan the skull deformities of patients in all directions and provide digital data [13,14]. Based on the data from the contralateral skull, orthopaedical surgeons can create a three-dimensional (3D) reconstruction model of the skull at the faulty site. Biomaterials can be created and produced into scaffolds for skull deformities using the generated digital data [13,15]. Calcium phosphate bone cements [16], polymers [17,18], titanium alloy [19], CuS-PEG-PCL scaffold [20] and other materials are currently widely used in skull and bone deformities [21,22,23,24]. The defect position can be covered and protected by the skull defect repair scaffold. The scaffold should also have osteoinductive properties for simple integration with surrounding bone tissue, ensuring that the scaffold is stable and no gaps in the defect position exist [25]. The scaffold needs to have a porous structure that can promote cell adhesion and migration to trigger the integration of the scaffold and surrounding bone [26]. The 3D printing technology can be used to print scaffolds based on the digital data of skull defects. The 3D printing can not only be utilised to create scaffolds with the same appearance and defect location, but it can also be used to create porous scaffolds. A suitable pore structure is conducive to cell adhesion, proliferation, and osteogenic differentiation, thereby achieving bone tissue regeneration [27,28,29]. Although traditional skull repair materials have good biocompatibility, they generally have poor osteoinductive capability. The inclusion of bioactive substances to the material to improve the osteoinductivity of the skull defect repair scaffold is one way to improve its osteoinductive capabilities. The majority of these bioactive compounds are growth factors [30], drugs, extracellular matrix [31], inorganic materials [32], etc. The loop-based and Argonaute-incorporated (AGO-incorporated) virtual screening model has been successfully used to obtain small molecules specifically targeting miRNA–mRNA interactions to rescue bone phenotype in genetically modified mice [33].

Polyvinyl alcohol (PVA) has the characteristic features of being a nontoxic, hydrophilic, biodegradable, and biocompatible compound and is extensively used in biomedical research such as healing of wounds, delivery of the drugs and tissue engineering [34,35,36]. β-Tricalcium phosphate (β-TCP) possesses chemical characteristics that are similar to those of bone minerals and is biocompatible [37]. β-TCP is also a material with good osteoinductive capability. The β-TCP component can act as a bridge in the scaffold, guiding the ingrowth of new bone and bone integration [38,39]. Therefore, β-TCP, as an excellent inorganic material, has been widely used in the preparation of bone repair scaffolds. Qianming et al. prepared a β-TCP bone repair scaffold. The antibacterial peptides and bone morphogenetic protein-2 mimetic peptide attached to the surface of the scaffold via DOPA tag, which achieved a good effect of promoting the differentiation of cells to osteogenesis [40]. Roberto Guarniero et al. combined platelet-rich plasma (PRP) and β-TCP to repair rabbit bone defects. After 4 weeks of repair, the scaffold containing PRP promoted a great amount of bone consolidation [41]. The above results indicate that β-TCP is a good bone repair material, but its osteogenic capability is still insufficient; thus, bioactive molecules should be added to enhance the osteogenic capability of the scaffold [22,42,43]. Icariin (ICA) is the main bioactive component of Herba Epimedii, which has been used to treat bone and joint diseases for a long time [44,45]. ICA can upregulate the expression of alkaline phosphatase (ALP), *Runx2*, *Osx* and collagen I, thereby stimulating the differentiation of cells into osteogenesis [46]. Chao Shi et al. added ICA to an nHAP/CMCS/PLGA scaffold as bioactive molecules. The scaffold containing ICA achieved a good repair effect in the skull defect repair experiment on rats [47]. These findings show that ICA can be used as a bioactive molecule in bone repair materials to promote osteogenesis.

In this study, we used a direct-ink 3D printing technology to prepare a scaffold that can effectively promote the repair of skull defects. The main components of the scaffold included β-TCP and bioactive molecules ICA. The bioprinting ink containing β-TCP and ICA was prepared using a polyvinyl alcohol (PVA) solution. The composition, hydrophilicity, mechanical properties, ICA release, cell adhesion, and ALP level of the scaffold were characterised in vitro. The bone repair capability of the scaffold was evaluated in vivo using the model of rat skull defect.

## 2. Materials and Methods

### 2.1. Materials

PVA was purchased from Sinopharm Chemical Reagent Co., Ltd. Haidian District, Beijing, (China). Β-TCP was purchased from Aladdin (Shanghai, China). ICA, Cell Counting Kit-8 (CCK-8), 4′,6-diamidino-2-phenylindole (DAPI), β-glycerol phosphate disodium salt pentahydrate, and vitamin C (VC) were purchased from Solarbio (Beijing, China). Dulbecco’s Modified Eagle Medium (DMEM) was obtained from Hyclone, 1725 S. HyClone Rd. Logan, Utah 84321 United States (USA). Foetal bovine serum (FBS) was purchased from Zhejiang Tianhang Biotechnology Co., Ltd. Zhejiang, (China). ALP assay, phalloidine and calcein/propidium iodide (PI) kits were purchased from Shanghai Beyotime Biotechnology Co., Ltd. Shanghai, (China).

### 2.2. Preparation and Characterisation of 3D-Printed Scaffolds

A total of 8 g of PVA was added to 100 mL of distilled water and heated in a water bath at 100 °C for 2 h until the PVA was completely dissolved to obtain 8% (*w*/*w*) PVA glue. Exactly 0.4 g of ICA was directly added to the 100 mL of PVA glue and stirred evenly. Then, 100 g of β-TCP powder was added and mixed for 10 min to prepare the printing ink. The mass ratio of each material was PVA glue:β-TCP:ICA = 50:50:0.2. A biological 3D printer (3D Creator 1.2, Ub biotech, China) was used for scaffold printing. The printing ink was filled into a 2 mL syringe for printing scaffolds. Solidworks 2017 was used to design the scaffold dimensions. The 10(L) × 10(W) × 10(H) mm^3^ scaffolds were used in an in vitro assay, and 8(⌀) × 2(H) mm^2^ scaffolds were used in the repair of the rat skull defect. Simply, 3D software was used to set up the printing parameters at a speed of 6 mm/s and a fill rate of 50%. The scaffold is naturally air-dried in a ventilated place for 24 h after the printing is completed and sufficient moisture has been evaporated to obtain a completed stent sample. The samples were sterilised by UV radiation before each test and stored in a centrifuge tube at room temperature.

The printed scaffolds were dried in the air for 24 h to obtain a PVA/β-TCP/ICA named as PTI scaffold. The PVA/β-TCP named as PT scaffold could be obtained without adding ICA when preparing the paste. All scaffolds were sterilised by ultraviolet radiation for 30 min.

The surface morphology and microstructure of the PT and PTI scaffolds were observed by a field emission scanning electron microscope (SEM) (S-4800, Hitachi, Tokyo, Japan). The chemical composition of the scaffolds was determined by Fourier transform infrared spectroscopy (FTIR, Perkin Elmer, FTIR-2000, Waltham, MA, USA). Images under the microscope were captured, and the analysis software program ImageJ was used to measure and calculate the pore size of scaffolds. Three scaffolds were selected for each group, and three holes were randomly selected for the measurement on each scaffold (*n* = 9). The porosity (Φ) (*n* = 5) of the scaffold was measured by the liquid displacement method, and the calculation formula is as follows:

$$\Phi =\frac{\left[\left(Wpse-Wps\right)-\left(Wpr-Wp\right)\right]/\rho e}{Vp-\left(Wpr-Wp\right)/\rho e}\times 100\%$$

where *Wps* is the total weight of the pycnometer with the dry scaffold, *Wpse* is the total weight of the pycnometer with the dry scaffold filled with ethanol, *Wpr* is the weight obtained by removing the scaffold and measuring the residual ethanol and pycnometer, and *Wp* is the dry pycnometer weight. *Vp* is the volume of the pycnometer, and *ρe* refers to the current density of ethanol at room temperature [48]. A contact angle meter (DSA10, Kruss, Hamburg, Germany) was used to measure the water contact angle of the material to evaluate its hydrophilicity [49]. A universal testing machine (Instron 1121, Instron, High Wycombe, UK) was used to test the mechanical properties of the samples, including their elastic modulus and compressive strength. The compression speed was set to 2 mm/min, and the loading weight was 5 kN [50].

### 2.3. ICA Release Characteristics

Different concentrations of ICA solutions were measured with the absorption value of 270 nm, and the standard curve fitting was performed using Origin 2017 software. The PTI scaffold was cut into small pieces (0.1 g) and placed in a 1 mL phosphate-buffered saline (PBS) solution for ICA release. The release condition was set at 50 rpm and 37 °C. At the time points of 1, 3, 5, 10, 20, 30, 45, 60, and 80 days, a 500 µL release solution was pipetted for ICA concentration determination. Then, 500 µL of fresh phosphate-buffered saline (PBS) was pipetted back into the release tube.

### 2.4. In Vitro Cell Experiments

Preosteoblast MC-3T3-E1 cells (the passage number was three) were cultured in a DMEM medium containing 10% (*v*/*v*) FBS under 5% CO_2_ at 37 °C, and the medium was changed every two days. The MC-3T3-E1 cells were seeded on scaffolds at a density of 2 × 10^4^ cells per well. At time points of 1, 3, 5, and 7 days, the proliferation ability of cells on the scaffold was measured using the CCK-8 kit. On day 3, the cells were stained using a phalloidine and calcein/PI kit.

For the in vitro osteogenic activity test of the scaffolds, the ALP activity test method was used. MC-3T3-E1 cells were seeded in a 12-well plate containing scaffolds at a density of 2 × 10^4^. After 48 h of culture, the medium was replaced with an osteogenic induction medium (10 mM β-sodium glycerophosphate and 50 mg/mL VC); then, half of the medium was changed every day until day 7 and 14 as described previously [22,51]. The cell culture medium was removed, and the cells were washed with PBS 3 times. Then, a 500 µL radioimmunoprecipitation assay cell lysis solution was added to each well, frozen, and thawed 3 times at −80 °C. After lysing the cells, the supernatant was obtained by centrifugation (13,000× *g*/10 min/4 °C), and the ALP content in cells was measured using an ALP assay kit (*n* = 3). The ALP activity was expressed in units of denitrification enzyme activity.

Similarly, the human vascular endothelial cells, EA.hy926, purchased from the Chinese Academy of Sciences Cell Bank, were cultured in endothelial growth medium-2 (EGM-2; Lonza), Basel (Switzerland) containing 10% foetal bovine serum in a 5% CO_2_ humidified incubator following the prescribed protocol [52]. The toxicity of scaffolds to EA.hy926 cells, on the proliferation, on migration, and on the expression of VEGF in EA.hy926 cells were analysed as previously described with slightly modifications [52,53,54]

### 2.5. In Vivo Animal Experiments

Fifty male Sprague Dawley rats (10 weeks old) weighing 250–300 g were randomly selected as experimental animals as previously reported [55]. The scaffold’s ability to mend bone was tested using a rat model of a skull defect. The defect site without scaffold was set as the defect group (DEF). An 8 mm diameter trephine was drilled in the middle of the parietal bone to create an 8 mm round defect, taking care to avoid damaging the sagittal sinus and dura mater. An 8(⌀) × 2(H) mm^2^ scaffold was placed at the round defect, and the wound was sutured. The animals were sacrificed after 4, 8, and 12 weeks to evaluate the effect of the scaffold on bone defect repair [21]. All animal studies were conducted in accordance with the principles and procedures outlined in the “Regulations for the Administration of Affairs Concerning Laboratory Animals”, approved by the National Council of China on 31 October 1988, and “The National Regulation of China for Care and Use of Laboratory Animals,” promulgated by the National Science and Technology Commission of China, on 14 November 1988. Decree No. 2. Protocols were approved by the Committee of Jilin University Institutional Animal Care and Use.

### 2.6. Micro-CT and Histological Evaluation

Skulls at different time points were scanned using micro-CT (Micro-CT35, Scanco Medical AG, Switzerland) to evaluate the effect of bone repair. The region of interest was selected at the 8 mm diameter defect-implantation site, and the bone volume over tissue volume (BV/TV) was measured (*n* = 3). The skull samples were decalcified with 10% ethylenediaminetetraacetic acid, embedded in paraffin, and sliced along the coronal plane in the defect-repair area. The sections were stained with haematoxylin eosin (H&E) and Masson’s trichrome (MT), observed, and photographed under a microscope.

### 2.7. Statistical Analysis

All the data in this study are presented as the mean and standard deviation for repeated trials. A one-way analysis of variance was used to evaluate the statistical significance of the differences between groups. *p* < 0.05 was considered significant in all tests.

## 3. Results

### 3.1. Characterisation

**Figure 1:**
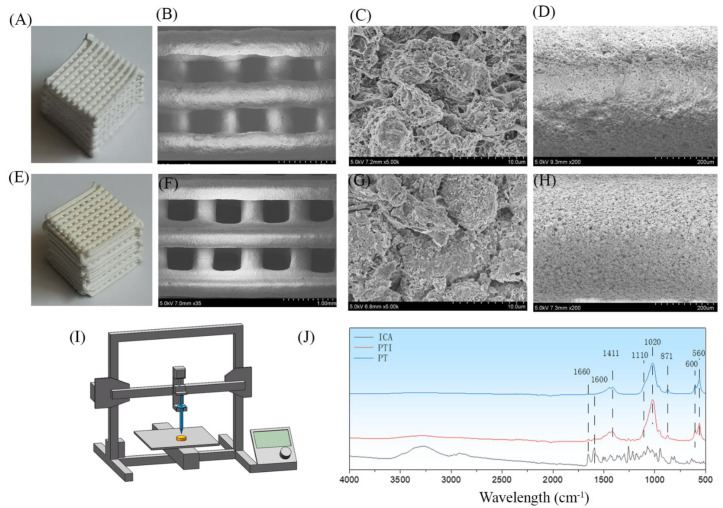
Physical appearance of scaffolds. Macroscopic photos and SEM micrographs of the PT (**A**–**D**) and PTI (**E**–**H**) composite scaffolds. High-resolution images of each category are provided as Supplementary Materials, File S1 (**I**). Three-dimensional printer schematic (**J**). FTIR was used to determine the surface chemical property of the scaffolds.

The physical appearance of the 10(L) × 10(W) × 10(H) mm^3^ scaffold was a 3D grid-like structure stacked by layers of filaments, with regular shapes and uniform pore diameters (Figure 1A,E). The PVA/β-TCP, onwards as PT scaffold, was white overall, whereas the PVA/β-TCP/ICA, onwards as PTI scaffold, was light yellow because of the addition of ICA. The microstructure of the scaffold was analysed by SEM. The pore size of the scaffold was relatively uniform, and the scaffold surface presented a relatively rough structure. In addition, small pores could be observed. Based on the results of the SEM images, no significant difference was observed between the surface morphology of the PT and PTI groups (Figure 1B–D,F–H). FTIR was used to determine the surface chemical property of the scaffolds (Figure 1J). The ICA absorption band appeared at 1600–1660 cm^−1^ (C=C and C=O). The typical absorption band of ICA (1600–1660 cm^−1^) can be observed on the PTI scaffold, suggesting the presence of ICA in the scaffolds.

**Figure 2:**
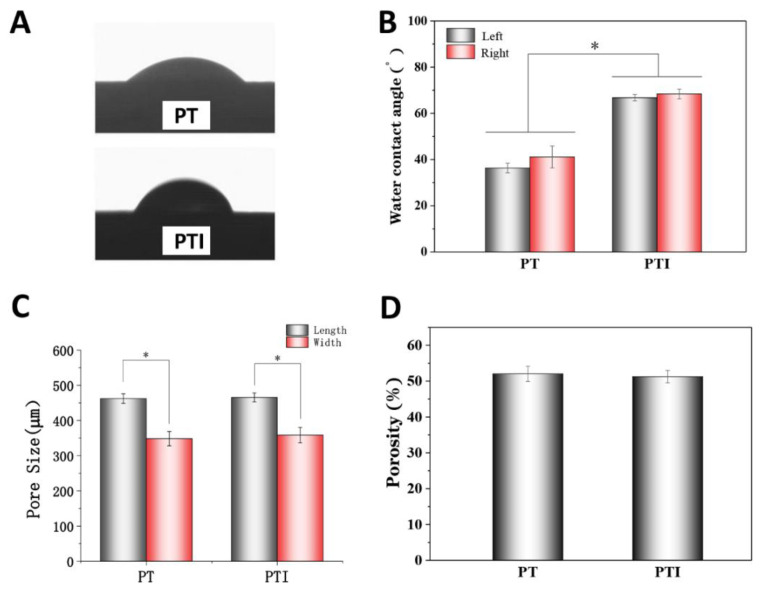
Water contact angle, pore size, and porosity of PT and PTI scaffolds. (**A**,**B**) Water contact angle, (**C**) average pore size, and (**D**) porosity of the two groups of scaffolds (*n* = 3, * *p* < 0.05).

The water contact angle experiment was used to measure the hydrophilicity of PT and PTI scaffolds. The water contact angles of the left and right sides of the PTI scaffold were 66.8° ± 1.4° and 68.4° ± 2.2°, respectively, which were significantly higher than those of the PT scaffold (36.3° ± 2.0° and 41.1° ± 4.8°) (*p* < 0.001). This finding may be related to the poor solubility of ICA in water (Figure 2A,B). The pore of the PT scaffold was 462 ± 13 × 368 ± 20 µm^2^, which was not significantly different from that of the PTI scaffold (466 ± 12 × 358 ± 21 µm^2^) (*p* > 0.05), as shown in Figure 2C. The porosities of PT and PTI were 52.03% ± 2.12% and 51.26% ± 1.71%, respectively, exhibiting no statistical significance (Figure 2D).

**Figure 3:**
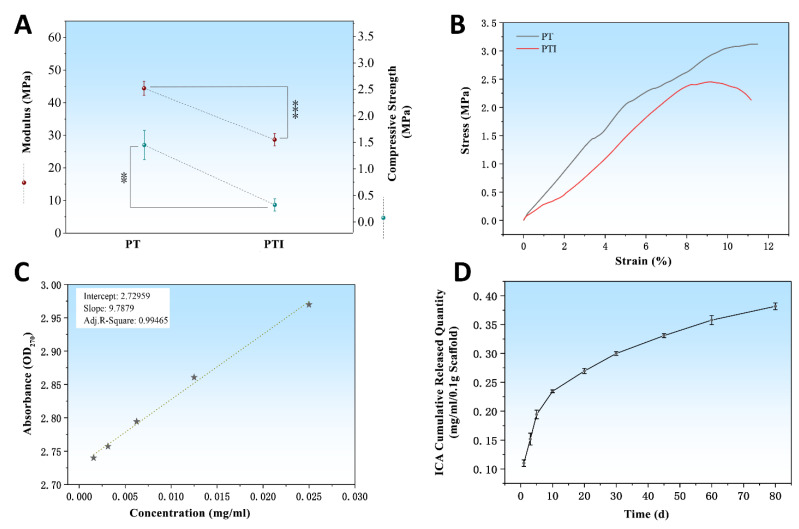
Mechanical properties of scaffolds and the release of ICA. (**A**) Mechanical properties of two groups of scaffolds including elastic modulus and compressive strength, (**B**) stress–strain curve showing the change trend of two groups of scaffolds after being stressed, (**C**) standard curve for ICA, and (**D**) the ICA release behaviour of PTI scaffold within 80 days. * *p* ≤ 0.05, ** *p* ≤ 0.01, *** *p* ≤ 0.001.

The elastic modulus and compressive strength of the scaffolds were investigated. The elastic modulus of the PT scaffold was 44.42 ± 2.11 MPa, which was significantly higher than that of the PTI scaffold (28.67 ± 1.88 MPa) (*p* < 0.001) as shown in Figure 3A. In addition, the compressive strength of the PT scaffold (1.45 ± 0.28 MPa) was greater than that of the PTI scaffold (0.32 ± 0.12 MPa) (*p* < 0.01). Combined with the stress–strain curve as shown in Figure 3B, we concluded that the compressive strength of the PT scaffold was significantly reduced after the incorporation of ICA. The standard curve of ICA was plotted using OD_270_ detection, provided in Figure 3C. Similarly, the ICA release kinetics of the PTI scaffold can be obtained. In the first 10 days, ICA showed the characteristics of burst release and the cumulative release of 0.23 mg/mL/0.1 g PTI scaffold, reaching 57.5% of the total ICA load as shown in Figure 3D. In the following 11–80 days, the release velocity of ICA lowered down significantly, and the cumulative release of ICA reached 0.38 mg/mL/0.1 g, accounting for 95% of the total ICA load.

### 3.2. Cell Compatibility and Adhesion Ability

**Figure 4:**
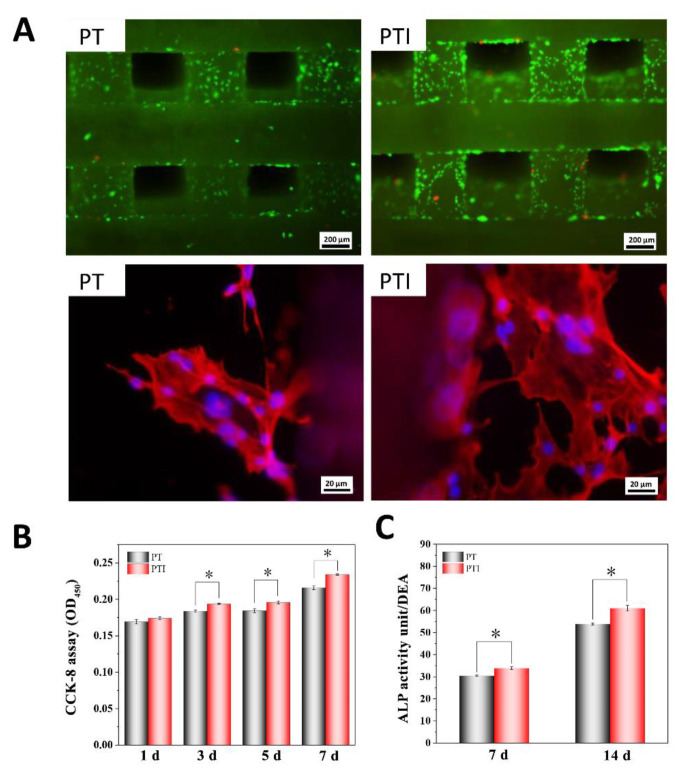
Cytocompatibility and osteogenic differentiation. (**A**) Living(green)/dead(red) cell and cytoskeleton/nucleus stain, (**B**) cell proliferation ability, and (**C**) ALP activity assay (*n* = 3, * *p* < 0.05).

The living cells were stained green with calcein AM, and dead cells were stained red with PI (Figure 4A). Calcein AM can penetrate the cell membrane and react in living cells to release the fluorescent molecule calcein. Therefore, calcein AM is usually used for the fluorescent staining of living cells. The cell compatibility of PT and PTI scaffolds was reasonable, and the number of cells on the surface of the PTI scaffold was significantly higher than that of the PTI scaffold group. For further observation of the growth and adhesion of cells, phalloidin can specifically bind to the F-actin in cells, and the cell skeletal structure can be displayed through fluorescence imaging. We stained and observed the cells with Alexa 546-labelled phalloidin and DAPI. As shown in Figure 4A, the cells were polygonal in shape and spread on a large area on the scaffold surface, and the nucleus was complete. Cytoskeleton proteins (F-actin) were ordered in the cell. These results indicated that the cell compatibility of PT and PTI scaffolds was good and showed no evidence of cytotoxicity.

The proliferative ability of cells growing on the scaffold was measured. On day 1, no significant difference was observed in the proliferation of cells grown on PTI and PT scaffolds. From day 3 to day 7, cell proliferation on the PTI scaffold was significantly higher than that on the PT scaffold. These results suggested that ICA can promote the proliferation of cells on the scaffold, Figure 4B.

### 3.3. ALP Activity

ALP activity is an important indicator of osteogenic differentiation. As shown in Figure 4C, after 7 and 14 days of cell growth, the ALP activity in the PTI group was significantly higher than that in the PT group. On days 7 and 14, the ALP activity in the PTI group increased by 11.24% and 13.32% compared with the PT group, respectively. These results suggested that ICA incorporated into the PT scaffold could promote the differentiation of cells into osteoblasts.

### 3.4. Toxicity of Scaffolds to EA.hy926 Cells

**Figure 5:**
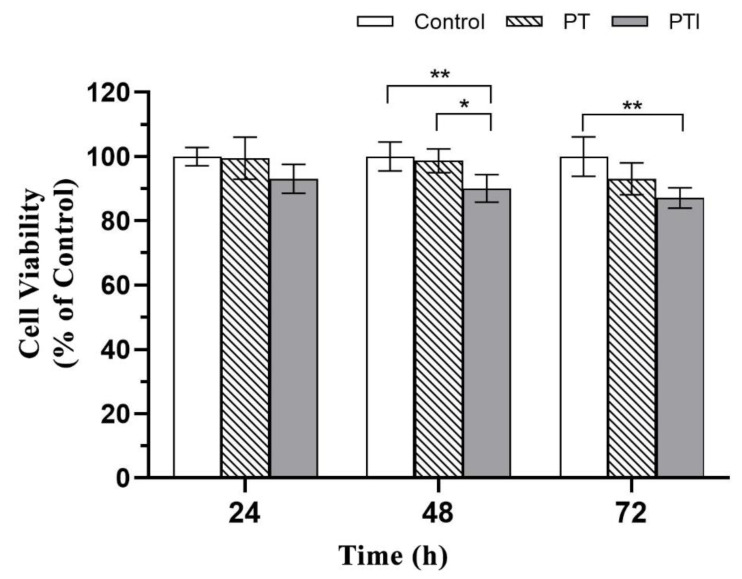
Cytotoxicity of scaffold extracts in each group. * *p* < 0.05, ** *p* < 0.01.

The cell survival rates of each group of scaffold extracts after coculture with EA.hy926 cells at different time points are shown in Figure 5. By a statistical analysis, after 24 h of culture, there was no significant difference in the cell survival rate among the groups (*p* > 0.05); after 48 h of culture, the cell survival rate of the PTI scaffold was lower than that of the PT scaffold (*p* < 0.05) and the control (*p* < 0.01), but there was no significant difference in the cell survival rate of the PT scaffold and the control; after 72 h of culture, the cell survival rate of the PTI scaffold was lower than that of the control (*p* < 0.05), but the cell survival rate of each group was greater than 80%. The GB/T 16886.5-2017 national standard stipulates that a decrease in cell activity greater than 30% is considered a cytotoxic reaction, so both groups of scaffolds did not have toxic effects on EA.hy926 cells and had good biocompatibility.

### 3.5. Effect of Scaffolds on the Proliferation of EA.hy926 Cells

**Figure 6:**
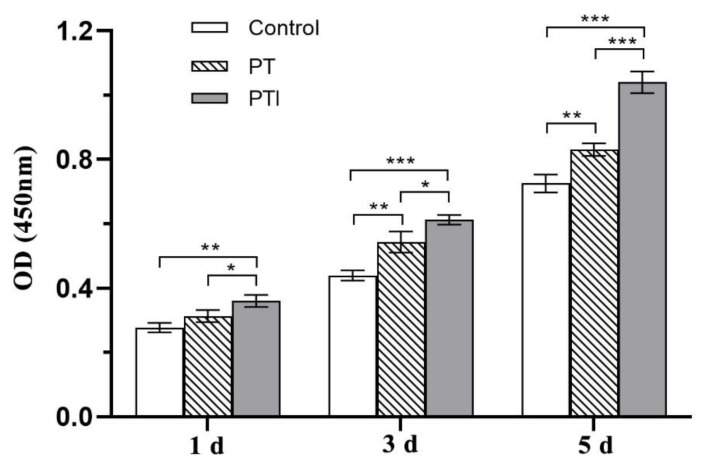
Cell proliferation activity of each group after coculture with scaffolds. * *p* < 0.05, ** *p* < 0.01, *** *p* < 0.001.

The effect of each scaffold on the proliferation of EA.hy926 cells was evaluated by the CCK-8. The cell proliferation was related to the OD value measured in each group, and the difference between the measured OD value and the OD value of the blank group was used as ordinate. The proliferation of cells after 1 d, 3 d, and 5 d of coculture with the scaffolds is shown in Figure 6. With the passage of time, the number of cells in each group increased. After a statistical analysis, the PTI scaffold showed a proliferation advantage over the control (*p* < 0.01) and PT scaffold (*p* < 0.05) after 1 d of coculture, but the proliferation advantage of the PT scaffold and control did not show significant differences (*p* > 0.05); after 3 d of coculture, the proliferation of the PTI scaffold was more prominent than that of the control (*p* < 0.001), and the cell proliferation of the PT scaffold began to be higher than that of the control (*p* < 0.01); after 5 d of coculture, the cell proliferation of the PTI scaffold was significantly higher than that of the control and PT scaffold (*p* < 0.001). It proved that both scaffolds could promote the proliferation of EA.hy926 cells, and the pro-proliferation ability of PTI was stronger than that of PT.

### 3.6. Effect of Scaffolds’ Leaching Solution on Migration of EA.hy926 Cells

**Figure 7:**
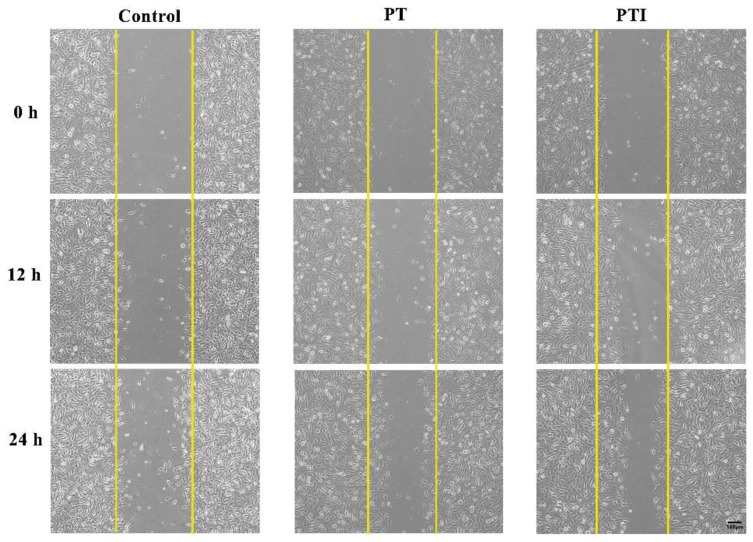
Scratch experiment to detect the effect of scaffolds’ leaching solution on the migration of EA.hy926 cells.

During vascular neogenesis, endothelial cells sprouted through the basement membrane, and merged with the sprouted endothelial cells of adjacent blood vessels through migration and proliferation to form a lumen structure, in which cell migration is an essential step. We used scratch experiment to detect the effect of the scaffolds’ leaching solution on the migration of EA.hy926 cells. As shown in Figure 7, the cell-free area in the figure is the scratch area. In order to reduce the effect of cell proliferation on the experimental results of cell migration, a serum-free medium was used for the culture. With the passage of time, the area of the scratched area gradually decreased, and it can be visualised that after stimulating the EA.hy926 cells with the scaffolds’ leaching solution, the number of cell migration increased, and the number of cell migration in the PTI scaffold was higher than that in the PT scaffold.

**Figure 8:**
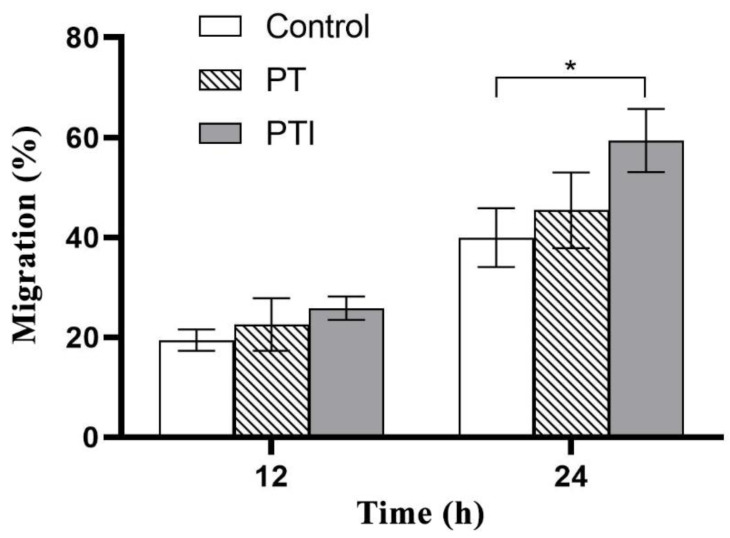
Quantitative analysis of cell scratching experiment. * *p* < 0.05.

A quantitative statistical analysis of the cell migration rate of each group was performed, and the results are shown in Figure 8. After stimulating the cells for 12 h, the difference in cell migration rate between the three groups was not statistically significant (*p* > 0.05), but after continuing stimulation until 24 h, the cell migration rate of the PTI was significantly higher than that of the control (*p* < 0.05), but there was no significant difference between the PTI and the PT scaffolds, as well as the PT scaffold and the control (*p* > 0.05). It proved that the leaching solution of the PTI scaffold could promote the migration of EA.hy926 cells and the effective molecule in the leaching solution to promote cell migration might be the ICA incorporated into the PTI scaffold.

### 3.7. Effect of Scaffolds on the Expression of VEGF in EA.hy926 Cells

**Figure 9:**
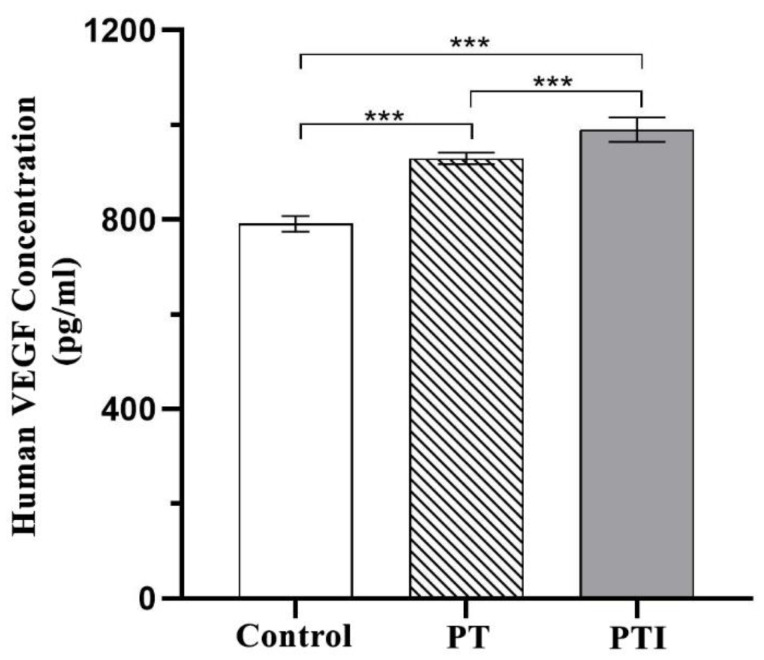
VEGF secretion levels in each group after coculture of cells with scaffolds *** *p* < 0.001.

As a key regulator of angiogenesis, VEGF can promote angiogenesis by stimulating the migration and proliferation of vascular endothelial cells to induce blood vessels to grow into areas with poor blood flow. The level of VEGF secretion in each treatment group was quantified by ELISA. The standard curve was made according to the instructions of the kit, and the content of VEGF in the cell culture supernatant of each group was converted according to the standard curve. The results are shown in Figure 9. After the scaffolds were inoculated with cells and cocultured for 72 h, the VEGF secretion level in the PTI was significantly higher than that in the PT and control groups (*p* < 0.001), and the VEGF secretion level in the PT scaffold was also significantly higher than that in the control (*p* < 0.001). It proved that both groups of scaffolds could upregulate the expression of VEGF in EA.hy926 cells, increase the secretion of VEGF, have a certain ability to promote angiogenesis, and the PTI scaffold was stronger than the PT scaffold.

### 3.8. In Vivo Osteogenesis of the Scaffold

**Figure 10:**
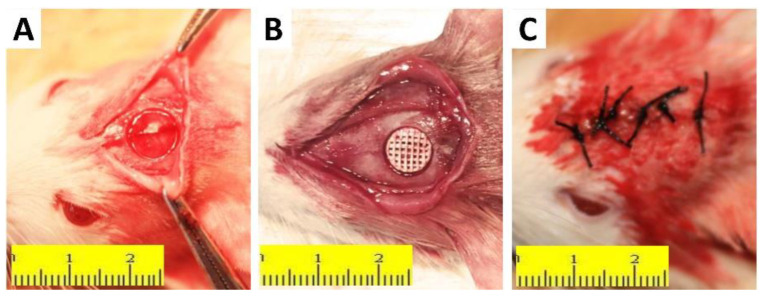
Osteogenesis of the Scaffold. Establishment process of rat large-sized skull defect model. (**A**) A round bone defect with a diameter of 8 mm was made in the skull of the rat. The brain and other structures were not damaged. (**B**) A stent with a diameter of 8 mm was inserted into the defect and the edges were almost identical. (**C**) Silk suture wound, good alignment, no obvious bleeding exudation was observed.

**Figure 11:**
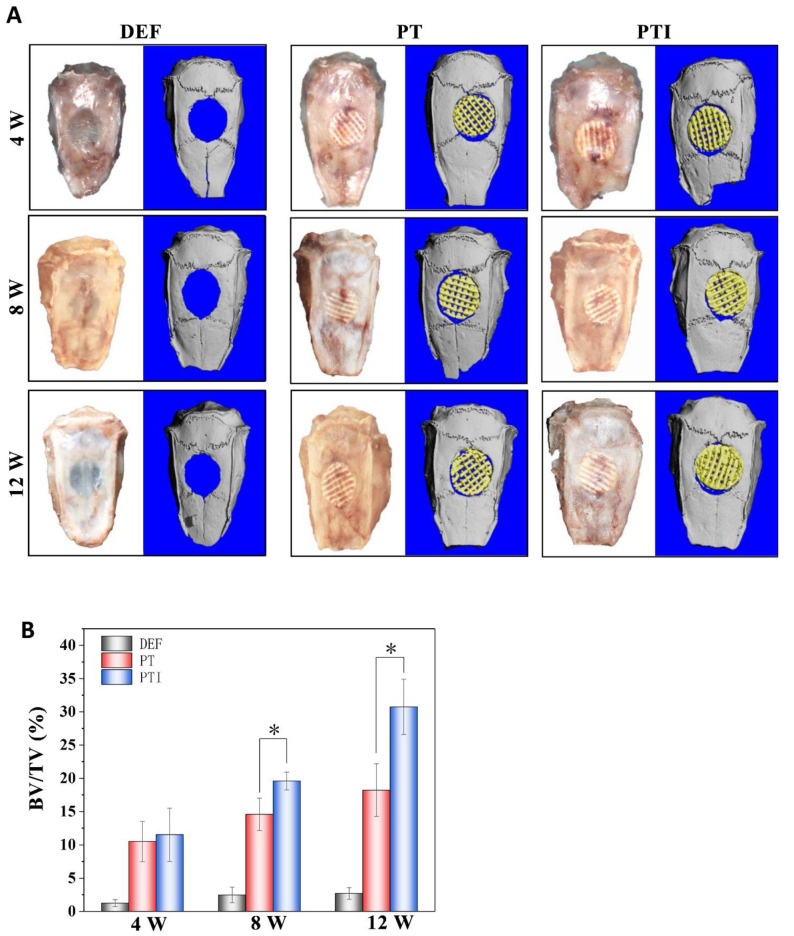
Micro-CT analysis and reconstruction results. (**A**) Three-dimensional reconstruction image of each specimen group; (**B**) BV/TV of defect site in PT and PTI groups at 4, 8, and 12 weeks (*n* = 3, * *p* < 0.05).

The establishment of the rat skull defect model is depicted in Figure 10. The size of the 8(Φ) × 2(H) mm^2^ scaffold fit tightly into the round defect of the rat skull. Animals were sacrificed at 4, 8, and 12 weeks after the operation, and all specimens were cut off from the rat skull for observation and assay. As shown in Figure 11, micro-CT was used to evaluate the repair effect of skull defects in different groups at various time points. From week 4 to week 12, no significant repair of the skull defect was performed on the DEF group. Digital photo results showed that the defect collapsed and was surrounded by fibrous connective tissue. The BV/TV in the DEF group did not increase significantly. These results indicated that the critical size skull defect cannot heal itself, and the skull defect needs to be repaired with appropriate scaffolds. In the PT and PTI groups, micro-CT results showed that the skull defect was filled with scaffolds, and the digital photo results revealed that the scaffolds connected with the skull and were firmly wrapped by fibrous tissue. Therefore, compared with the DEF group, the implantation of PT and PTI scaffolds at the defect site offered better protection of the nerve tissue and brain tissue inside the skull. As shown in Figure 11, a new bone formed along the scaffold surface in the PT and PTI groups at 8 weeks, and the effect of new bone formation in the PTI group was higher than that in the PT group. The BV/TV of PTI and PT groups were 14.56% and 19.58%, respectively. At 12 weeks, several of the pores of the scaffold were filled with new bone. The effect of new bone formation in the PTI group was significantly higher than that in the PT group, and this trend further increased at 8 weeks. The BV/TV of the PTI and PT groups reached 18.21% and 30.74%, respectively. These results suggest that the addition of ICA in the PT scaffolds can promote the formation of new bone.

**Figure 12:**
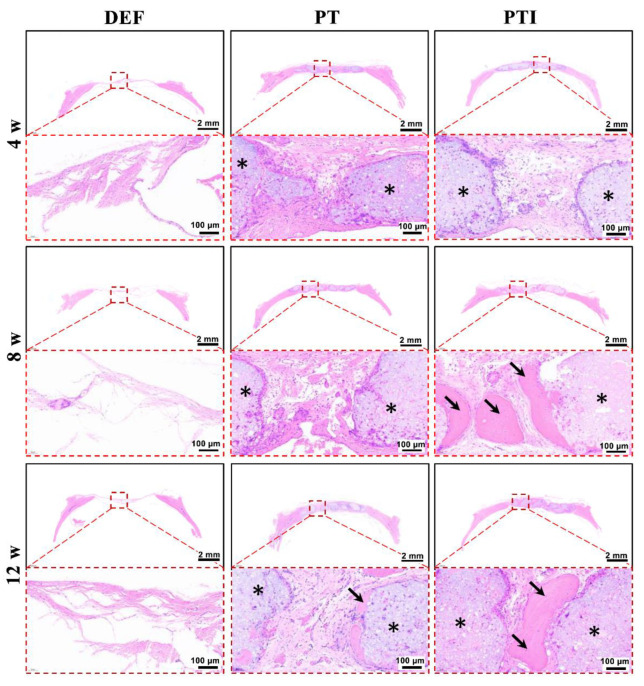
Microscopic appearance of tissue sections after H&E staining. Black stars indicate scaffolds, and black arrows denote new bone, where * is the part of the scaffold material shown after sectioning staining, while → refers to the new bone formed in the gap between the scaffolds.

**Figure 13:**
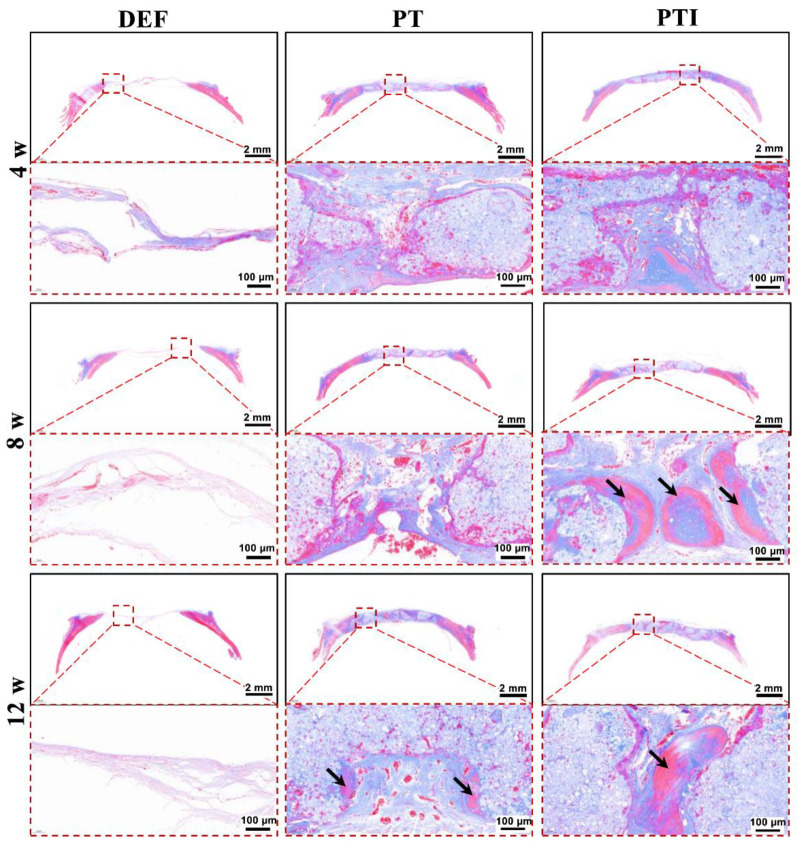
Microscopic appearance of tissue sections after MT staining; the arrows indicate new bone.

Histological analysis results of skull specimen were investigated by H&E staining (Figure 12) and Masson’s Trichrome, MT, staining (Figure 13). The DEF group presented a thin layer of fibrous connective tissue, which did not change significantly over time. The PT and PTI scaffolds (black stars) were securely encircled by fibrous connective tissue at 4 weeks, with a modest number of inflammatory cells surrounding the scaffold’s surface and no evidence of new bone formation (black arrows). Collagen deposition was greater in the PTI group than in the PT group. At week 8, no new bone formation was observed in the PT group, and a small number of inflammatory cells were still wrapped on the scaffold surface. However, the PTI group contained a large amount of new bone formation and no evident inflammatory cells. MT staining also revealed the formation of new bone. At weeks 12, the number of inflammatory cells significantly decreased, and a small amount of new bone formed along the surface of the scaffold in the PT group, but the proportion of new bone formation significantly increased in the PTI group. These H&E and MT staining results were consistent with the micro-CT images and BV/TV results in Figure 11.

## 4. Discussion

Clinically, skull defects need a suitable scaffold for filling and repairing. The skull has a thin and spherical structure, and autologous bone and allogeneic bone grafts cannot effectively repair large-sized skull defects [56]. Metal, inorganic, and polymer materials for skull defect repair have been widely developed and used to solve the problems faced by skull defects [57,58]. The skull defect of patients has a personalised shape and repairing it with mass-produced scaffolds is difficult. Three-dimensional printing processing technology brings a new direction for the preparation of personalised scaffolds of skull defects based on patient CT data [59]. According to different principles, 3D printing can be divided into fused deposition modelling (FDM) [60], stereo lithography appearance (SLA) [61], selective laser sintering (SLS) [62], etc. The temperature of the material during the FDM and SLS printing process is extremely high. Therefore, adding bioactive molecules to the printed scaffold is difficult. Although the processing temperature of SLA can be maintained at room temperature, the monomers and catalysts in the scaffold are difficult to remove. The printing technology used in this study was direct-ink 3D printing technology [63]. The printing ink was prepared using PVA, β-TCP, and ICA. PVA acted like glue, bonding other components together. In this condition, the ink can be stacked into a scaffold without heating. The bioactive molecule ICA in the scaffold can maintain its activity well. PVA and β-TCP materials were used by several research teams to prepare 3D-printed bone repair scaffolds. Wei Niu et al. used PVA and gelatine (Gel) as a glue to prepare a 3D printing ink containing β-TCP. The Gel–PVA–TCP scaffold was sintered at 1000 °C for 2 h to remove the glue and obtain a pure β-TCP scaffold [64]. In this study, only inorganic materials were left, and the bioactive molecules could not be combined. Gel was an excellent bioactive protein that promoted cell adhesion, but it only acted as a glue, and it was finally removed by sintering. The compressive strength of the pure β-TCP scaffold was between 3.47–3.82 MPa, whereas that of the scaffold prepared in our study was between 0.32–1.45 MPa (Figure 3A). Although the compressive strength of PT and PTI scaffolds was lower than that of the pure β-TCP scaffold, the elastic moduli of the PT and PTI scaffolds were 44.42 ± 2.11 and 28.67 ± 1.88 MPa, respectively, which were considerably lower than that of the pure β-TCP scaffold. The elastic modulus of the pure β-TCP scaffold was greater than 200 MPa [65]. These results proved that PT and PTI scaffolds were more flexible than pure β-TCP scaffolds. The area of the skull defect collided inevitably with an external force, and the flexible PTI scaffold could withstand the external force without being damaged. Although the 3D-printed scaffold of skull repair can be used to customise the appearance and internal structure of the scaffold, the scaffold should be provided with a strong osteoinductive capability. The main components of the scaffold in this study were PVA, β-TCP, and ICA. PVA served as an adhesive, β-TCP exhibited osteoconductivity capability, and ICA belonged to bioactive molecules that promoted bone formation at the defect site [40,66]. ICA was mixed with the 3D-printed bone repair scaffold through different methods, and a good repair effect was obtained. Ling Qin et al. used low-temperature 3D printing technology to prepare a β-PLGA/TCP bone repair scaffold containing ICA. 1,4-Dioxane was used to dissolve PLGA during the printing ink preparation process, and organic solvents were difficult to remove completely, implying a potential inflammatory response in vivo [22]. The scaffold printing process in this study did not use any solvents. Thus, no potential inflammatory reactions were caused by residual solvents. Living/dead cell and cytoskeleton/nucleus stain and cell proliferation assay results also proved that PTI and PT scaffolds had no evident cytotoxicity (Figure 4). ICA is stored at room temperature, whereas growth factors need to be stored at low temperatures to maintain activity. Therefore, the ICA used in this study was more suitable as a bioactive molecule to prepare the scaffold of the skull repair. In the rat skull defect animal model, the PTI scaffold was used to repair the skull defect. At 12 weeks after surgery, a large amount of new bone formed around the PTI scaffold, and no evident inflammation was observed during the entire repair period. A group of researchers have used the human skeletal-muscle-specific ssDNA aptamer (HSM01) and found that it preferentially interacted with human skeletal muscle cells in vitro. The in vivo study using tree shrews showed that the HSM01 ssDNA aptamer specifically targeted human skeletal muscle cells [67]. We also performed the additional experiments to evaluate the scaffolds effect on the human EA.hy926 cells. The cells migration assay was in accordance with other researchers [52], who used the icariin as an autophagy agent.

## 5. Conclusions

In this study, a scaffold for skull bone repair containing ICA was prepared by direct-ink 3D printing technology. The entire printing process was completed at room temperature without using a solvent, which can maintain the activity of the bioactive molecule ICA. In vitro results proved that the PTI scaffolds had good cell adhesion, cell compatibility, and osteogenic differentiation capabilities. The PTI scaffold also achieved good repair effects in the model of rat skull defect. The PTI scaffold has wide application prospects in the field of skull defect repair.

## Data Availability

Not applicable.

## Supplementary Materials

The following supporting information can be downloaded at: https://www.mdpi.com/article/10.3390/molecules27144535/s1, Figure S1. Physical appearance of scaffolds. Macroscopic photos and SEM micrographs of the PT (A, B, C and D) and of the PTI (E, F, G and H) composite scaffolds. High resolution images are provided.

## Sample Availability

Samples of the compounds are available from the authors on demand.
