# Supplementary material for: Engineered 3D-Printed Polyvinyl Alcohol Scaffolds Incorporating β-Tricalcium Phosphate and Icariin Induce Bone Regeneration in Rat Skull Defect Model
Source: Molecules. 2022 Jul 15;27(14):4535. doi: 10.3390/molecules27144535 (PMC9318678; doi:10.3390/molecules27144535)
Supplement: Supplementary file 1 [file molecules-27-04535-s001.zip › molecules-1781692-supplementary.pptx]

## Slide 1
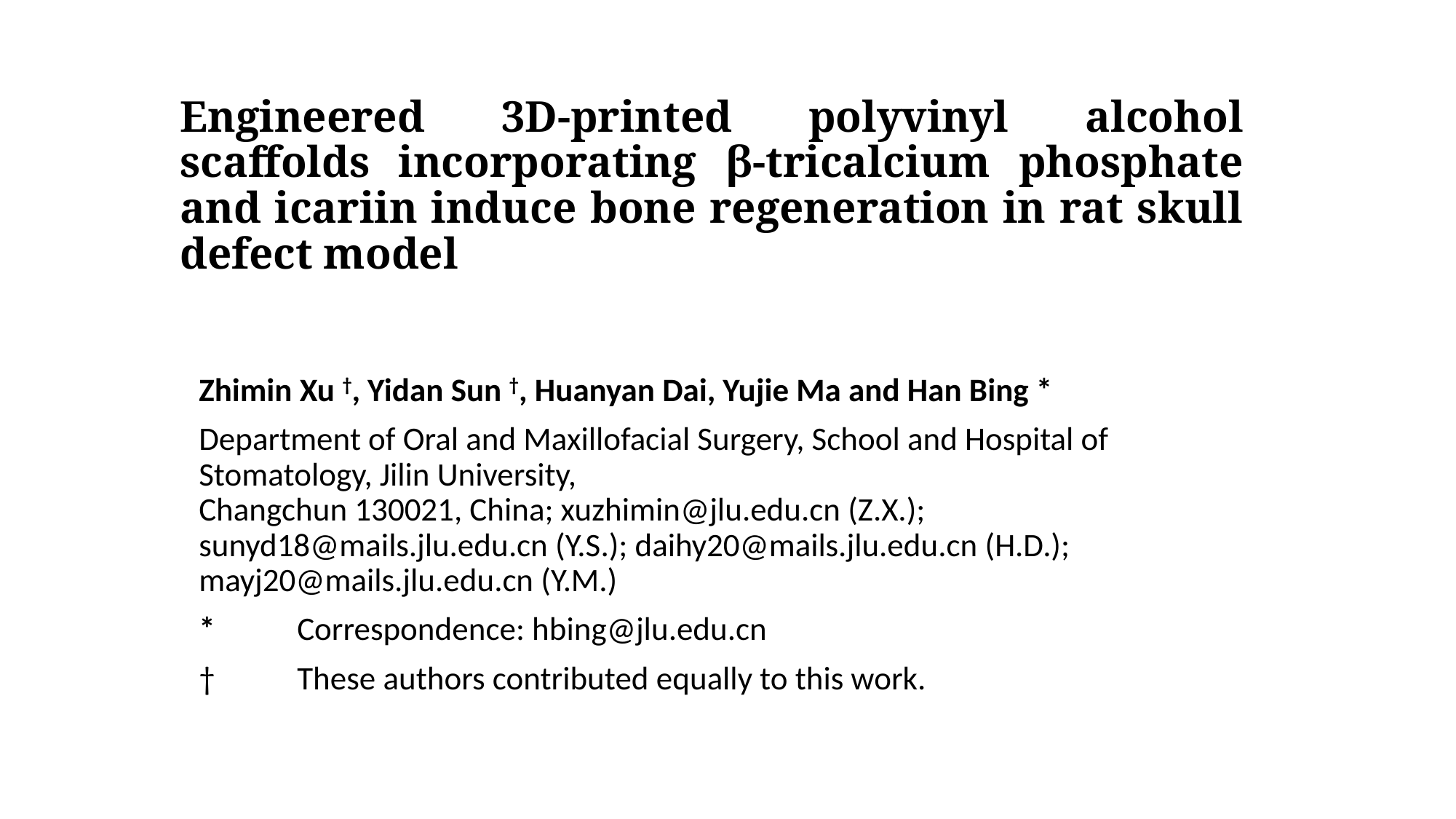

# Engineered 3D-printed polyvinyl alcohol scaffolds incorporating β-tricalcium phosphate and icariin induce bone regeneration in rat skull defect model
Zhimin Xu †, Yidan Sun †, Huanyan Dai, Yujie Ma and Han Bing *
Department of Oral and Maxillofacial Surgery, School and Hospital of Stomatology, Jilin University,
Changchun 130021, China; xuzhimin@jlu.edu.cn (Z.X.); sunyd18@mails.jlu.edu.cn (Y.S.); daihy20@mails.jlu.edu.cn (H.D.); mayj20@mails.jlu.edu.cn (Y.M.)
*	Correspondence: hbing@jlu.edu.cn
†	These authors contributed equally to this work.

## Slide 2
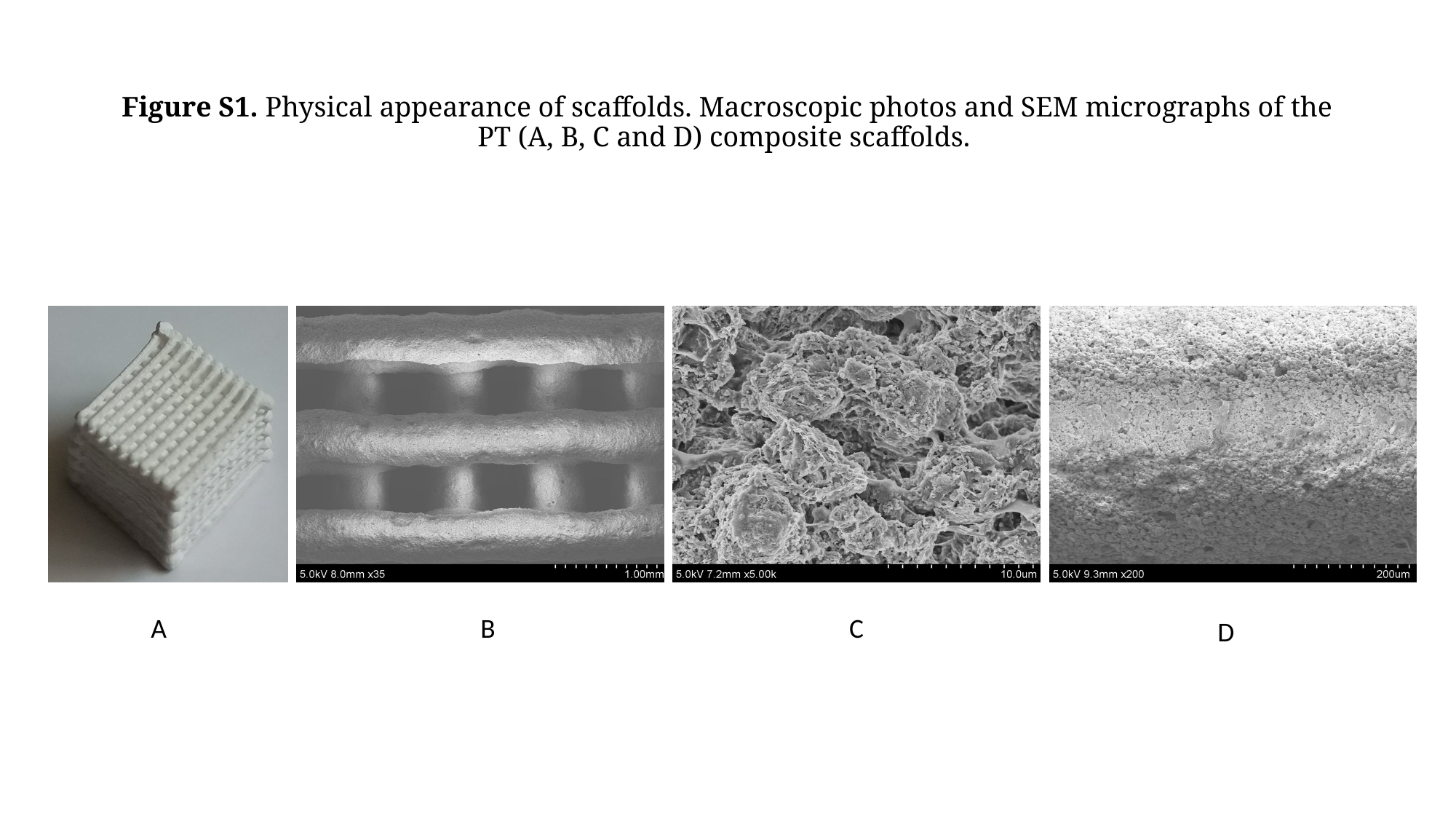

# Figure S1. Physical appearance of scaffolds. Macroscopic photos and SEM micrographs of the PT (A, B, C and D) composite scaffolds.
A
B
C
D
